# Neuroimaging glutamatergic mechanisms differentiating antipsychotic treatment-response

**DOI:** 10.1038/s41598-022-26702-0

**Published:** 2023-06-02

**Authors:** Elias D. Mouchlianitis, Lucy D. Vanes, Derek K. Tracy, Anne-Kathrin Fett, Daniel Joyce, Sukhi S. Shergill

**Affiliations:** 1 Institute of Psychiatry, Psychology and Neuroscience, De Crespigny Park, London, SE5 8AF UK; 2 School of Psychology, University of East London, Water Lane, Stratford, London, E15 4LZ UK; 3 West London NHS Trust, London, UB2 4SD UK; 4 Department of Psychiatry, University College London, London, W1T 7BN UK; 5 Department of Psychology, City, University of London, London, EC1V 0HB UK; 6 Oxford Health NHS Foundation Trust, Oxford, OX4 4XN UK; 7 Kent and Medway Medical School, Kent, CT2 7FS UK

**Keywords:** Cognitive neuroscience, Social behaviour, Biomarkers

## Abstract

Glutamatergic dysfunction is associated with failure to respond to antipsychotic medication in individuals with schizophrenia. Our objective was to combine neurochemical and functional brain imaging methods to investigate glutamatergic dysfunction and reward processing in such individuals compared with those with treatment responsive schizophrenia, and healthy controls. 60 participants played a trust task, while undergoing functional magnetic resonance imaging: 21 classified as having treatment-resistant schizophrenia, 21 patients with treatment-responsive schizophrenia, and 18 healthy controls. Proton magnetic resonance spectroscopy was also acquired to measure glutamate in the anterior cingulate cortex. Compared to controls, treatment responsive and treatment-resistant participants showed reduced investments during the trust task. For treatment-resistant individuals, glutamate levels in the anterior cingulate cortex were associated with signal decreases in the right dorsolateral prefrontal cortex when compared to those treatment-responsive, and with bilateral dorsolateral prefrontal cortex and left parietal association cortex when compared to controls. Treatment-responsive participants showed significant signal decreases in the anterior caudate compared to the other two groups. Our results provide evidence that glutamatergic differences differentiate treatment resistant and responsive schizophrenia. The differentiation of cortical and sub-cortical reward learning substrates has potential diagnostic value. Future novel interventions might therapeutically target neurotransmitters affecting the cortical substrates of the reward network.

## Introduction

Excess striatal dopamine is a hallmark of psychotic illnesses. The aberrant salience model^1^ postulates that this results in imprecise encoding, reward processing deficits, and attribution errors without appropriate contextual relevance. Clinically, this leads to the development of delusions to explain such associations. This is attenuated by antipsychotic medications^2^ whose effectiveness is due to antagonistic binding to post-synaptic D2 receptors^3^. However, approximately 30% show few or no gains from currently available treatments^4^. Data from dopamine depletion and positron emission tomography (PET) studies show that some individuals with refractory illness can have normal dopaminergic functioning^5–8^, which may explain their medication failure. Rather, a primarily glutamatergic dysfunction may be underpinning symptomatology, as evidenced by recent proton magnetic resonance spectroscopy (1H-MRS) studies that report elevated anterior cingulate cortex (ACC) glutamate levels in both first-episode and chronic illness individuals. Further, work by our group has shown significant glutamatergic differences between treatment resistant and non-treatment resistant individuals with schizophrenia^9^. However, any clear link between glutamatergic dysfunction and antipsychotic treatment-resistance has hitherto not been neurobiologically demonstrated.

Striatal processing deficits leading to the development of psychosis might be perturbed by multiple pathways, not exclusively dopaminergic. The human reward network and optimal learning performance is predicated on intact functioning and interaction of both cortical and sub-cortical nodes^10,11^. Such cortical substrates are densely populated by interneurons innervated by *N*-methyl-D-aspartate receptors (NMDAR)^12^. Complex reward encoding and learning processes—especially under a social interaction context to infer motivational salience—require the integration of reward and sensory information within the ACC, dorsolateral prefrontal cortex (DLPFC) and parietal association cortex (PAC) (10).

Taking the above together, we hypothesized that for treatment-resistant individuals, symptomatology is primarily driven by reward processing deficits stemming from glutamatergic dysfunction in key cortical substrates involved in reward processing. To investigate this, we investigated reward processing, tested through a social reward learning task, using a multi-modal brain imaging approach by combining functional magnetic resonance imaging (fMRI) and proton magnetic resonance spectroscopy (1H-MRS) to measure glutamate levels from the ACC. The ACC was selected based on previous evidence for elevated glutamate levels in a sample of chronic treatment-resistant patients^9^. We hypothesised that in treatment-resistant individuals glutamate would modulate reward processing cortical substrates, whereas those who were treatment-responsive would have an associated aberrant reward signaling in the striatum.

## Patients and methods

### Participants

We recruited 42 patients with an existing diagnosis of schizophrenia (based on ICD-10 1992 criteria) from the South London and Maudsley (SLaM) National Health Service (NHS) Trust. Patients were recruited via liaison with the responsible care coordinators in the Trust. 18 healthy controls were recruited via local advertising in the same area of South London. Recruitment took place between January 2015 and May 2016 and study assessments were conducted by a trained member of the research team (LDV). The patient cohort included 21 who were treatment-resistant, defined as having at least two prior drug trials of 4–6 weeks duration with no clinical improvement, persistence of illness for longer than five years with no period of good social or occupational functioning, and persistent psychotic symptoms as defined as a score of at least 4 (moderate) on at least two positive symptom items of the Positive and Negative Syndrome Scale (PANSS^13^). The remaining 21 patients fulfilled criteria for being in symptomatic remission, as defined by a score of 3 or less on all items of the PANSS^14^ and these symptoms having been stable for at least 6 months^15^.The two patient groups did not differ in regard to age, sex, duration of illness, and antipsychotic dosage (Table 1). Current clozapine use was an exclusion criterion for all patients, as it has been shown to attenuate glutamate levels in treatment-resistant schizophrenia patients^16^.

**Table 1:** Means (M) and standard deviations (SD) of demographic and clinical variables per group: WASI, Wechsler Abbreviated Scale of Intelligence; NS-SEC, National Statistics Socio-economic Classification; CPZ, Chlorpromazine; PANSS, Positive and Negative Symptom Scale.

	Controls (n = 18)		Responsive (n = 21)		Resistant (n = 21)		Group statistics	
	*M*	*SD*	*M*	*SD*	*M*	*SD*		
							*χ*^*2*^(2)	*P*
Male/female	12/6		14/6		14/6		1.18	.555
Smokers (%)	24		67		62		13.0	< .001
							*F*(2,57)	*P*
Age	40.6	9.4	41.3	10.4	41.5	10.6	0.26	.77
WASI	114.6	12.1	91.86	14.8	97.1	16.4	16.8	< .001
NS-SEC	3.05	1.52	3.74	1.88	3.39	1.76	0.42	.61
							*t*(40)	*P*
Onset age (years)			27.7	6.2	26.0	7.7	0.80	.431
Illness duration (years)			14.1	10.1	15.5	8.8	0.46	.650
CPZ equivalents			280.3	147.1	383.5	236.5	1.67	.103
**PANSS score**								
Positive symptoms			10.7	2.1	20.5	3.1	12.10	< .001
Negative symptoms			13.1	4.6	19.5	4.6	4.08	< .001
General symptoms			23.6	5.1	34.9	9.2	5.91	< .001
Total score			46.9	10.3	76.2	10.6	9.14	< .001

Intelligence quotient was measured with the two-item Wechsler Abbreviated Scale of Intelligence (WASI). We did not have an explicit criterion for intellectual ability; however, all participants had an IQ > 75 as assessed by the WASI. Chlorpromazine (CPZ) equivalent doses of medications were calculated using conversion tables^17^. Exclusion criteria for all subjects were a history of neurological illness, current major physical illness, and drug dependency over the last six months. Exclusion criteria for healthy controls were a history of psychiatric illness and a first-degree relative having suffered from a psychotic illness. All subjects had normal hearing and normal or corrected-to-normal vision. Ethical approval was provided by the London Camberwell St Giles Research and Ethics Committee. All participants provided informed written consent and were compensated for their time and travel. The present research, and all methods contained within, was conducted in accordance with the Declaration of Helsinki.

### fMRI task

**Figure 1:**
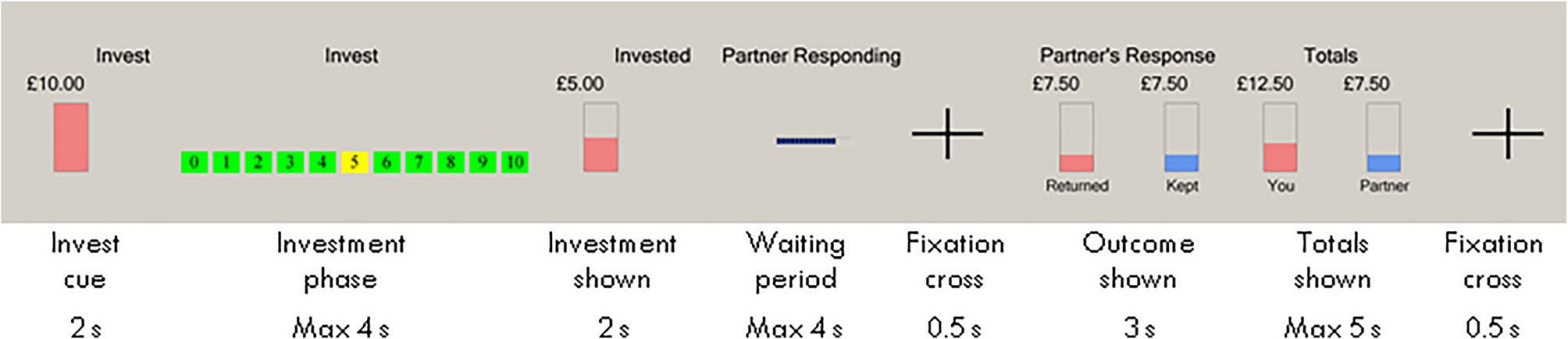
Experimental set-up of a trust game round.

The trust game was a modified version of a previous multi-round trust game^18^, as described in Gromman et al.^19^ (Fig. 1). In the present study participants only played role of the investor in the cooperative version of the game, and were explicitly informed that they would be playing with a computer, deciding the amount of money, (£1–10), to share with it. The repayment percentage was calculated probabilistically based on current and previous investment. Ideally, participants would maximise their returns by identifying the cooperative playing style of the player and develop trust. This form of implicit reward learning involves both decision making and risk calculation, while it engages both striatal and cortical substrates of the reward processing network^20^.

### Analysis of demographic, clinical, and behavioural data

Demographic, clinical and behavioural data were analysed using a *χ*^2^, one-way analysis of variance (ANOVA; P < 0.05) and two-sample t-tests (two-tailed, P < 0.05) as appropriate. Behavioural data were analysed in terms of investment for the first trial and mean investment across all 20 experimental trials.

### fMRI analysis

FMRI data processing was carried out using a general linear model as implemented in FEAT (FMRI Expert Analysis Tool) Version 6.00, part of FSL (FMRIB's Software Library, www.fmrib.ox.ac.uk/fsl). Functional and structural brain images were extracted from non-brain tissue using FSL’s brain extraction tool (BET), and EPI images were realigned using MCFLIRT to correct effects of head motion. A 100-s temporal high-pass filter was applied, and data were spatially smoothed using a Gaussian kernel of 6 mm FWHM.

For the first-level analysis, the investment and repayment phases of the real and control trials of the task were modelled separately. Each regressor was modelled with a delta function of zero duration and convolved with a canonical haemodynamic response function and its temporal derivative. Six standard motion parameters as well as a motion artefact confound matrix, which identified motion-corrupted volumes, were added as regressors of no interest. Volumes detected as corrupted were calculated by DVARS metric^21^, as implemented by FSL Motion Outliers in FSL (https://fsl.fmrib.ox.ac.uk/fsl/fslwiki/FSLMotionOutliers). Contrasts of interest for each subject were created by comparing mean BOLD signal of investment and repayment trials to their respective control trials.

### ^1^H^-^MRS acquisition

All scans were acquired on a General Electric (Milwaukee, Wisconsin) 3 Tesla HDx magnetic resonance system as detailed previously (Egerton et al. 2012). ^1^H-MRS spectra (PRESS—Point RESolved Spectroscopy; TE = 30 ms; T*r* = 3000 ms; 96 averages) were acquired using the standard GE PROBE (proton brain examination) sequence, which uses a standardized chemically selective suppression water suppression routine. Shimming was optimized, with auto-prescan performed twice before each scan. An initial localizer scan was followed by acquisition of structural images, including an axial 2D T2-weighted fast spin echo scan and an axial fast fluid-attenuated inversion recovery scan. The anterior cingulate cortex voxel was prescribed from the midline sagittal localizer, with the centre of the 20 × 20 × 20 mm voxel placed 13 mm above the genu of corpus callosum perpendicular to the AC–PC line.

### ^1^H^-^MRS quantification and analysis

Data were analyzed using LCModel version 6.3^22^ (http://s-provencher.com/lcmodel.shtml). A standard basis set of 16 metabolites was used (comprising L-alanine, aspartate, creatine, phosphocreatine, GABA, glucose, glutamine, glutamate, glycerophosphocholine, glycine, myoinositol, L-lactate, *N*-acetyl aspartate, *N*-acetylaspartylglutamate, phosphocholine, taurine), including simulated lipids and macromolecules as part of LCModel basis set that was acquired with the same field strength (3 T), localization sequence (PRESS) and echo time. Model metabolites and concentrations employed in the basis set are fully detailed in the LCModel manual (http://www.s-provencher.com/pub/LCModel/manual/manual.pdf). Metabolite concentration estimates were expressed in ratio to total creatine (Cr) which is calculated as Cr plus phosphocreatine (PCr) within LCModel. NAA was expressed as *N*-acetyl aspartate plus *N*-acetylaspartylglutamate, and choline as glycerylphosphorylcholine plus phosphocholine. Only metabolite concentration estimates associated with Cramer-Rao lower bounds (CRLB) < 20% as reported by LCModel were included in the analysis. Additionally signal-to-noise ratio (S/N) ≥ 10 and linewidth of FWHM < 0.1 ppm was required for inclusion.

### Region of interest analysis

Given previous evidence for normal striatal dopaminergic function in treatment-resistant patients^7^, we investigated whether striatal BOLD signaling would differ between the three groups during the trust task. We defined a priori the striatal region of interest (ROI) using a 6 mm radius sphere around a peak coordinate [MNI: x =  ± 12, y = 12, z = 4; Fig. 4A] taken from a previous study using the same task, that showed a reduction in caudate activation during the investment phase in stable schizophrenia patients^19^.

### Integration of fMRI and ^1^H-MRS data

The relationship between the BOLD signal to investment and repayment trials and Glu/Cr levels in the ACC was investigated by entering the individual Glu/Cr values as covariates in an analysis of variance design with the fMRI contrast images (investment vs control; repayment vs control). Glutamate × BOLD signal interactions were first partitioned by each group (treatment-resistant; treatment-responsive; controls) and then group × glutamate × BOLD signal interactions were assessed within the same design matrix by performing mixed-model analysis as implemented in FSL (FLAME1 + 2) with automatic outlier de-weighting. IQ, medication dose and illness duration were included as nuisance covariates. Group contrast statistic images were thresholded using clusters determined by Z > 3.1 and a whole-brain corrected cluster significance threshold of P = 0.05. For completeness, using the same threshold, we conducted whole-brain analyses on the main effects of task (investment and repayment trials) and the main effect of group (treatment-resistant vs treatment-responsive vs healthy controls) without including glutamate as a covariate.

## Results

### Data availability

The datasets used and/or analysed during the current study available from the corresponding author, Dr Elias Mouchlianitis, on reasonable request. The full dataset contain data that are still unpublished.

### Behavioural and 1H-MRS analysis

Analysis of the behavioral results showed that there were no differences between the groups in the initial investment (Ps > 0.4). There was a significant difference between the groups in mean investment across the 20 rounds of the game, *F*(2,57) = 3.16, *P* < 0.05 (see Table 2). Compared to controls, both schizophrenia groups showed significant reductions in mean investment: treatment-responsive versus controls [*T*(37) = 2.77, *P* < 0.01, *d* = 0.91]; treatment-resistant vs controls [*T*(37) = 2.11, *P* < 0.05, *d* = 0.69], while the patient groups did not differ from each other [*T*(40) = 0.97, *P* > 0.3, *d* = 0.31]. There were no differences in glutamate levels between the groups or in any other metabolites, all *P*s > 0.2.

**Table 2:** Means (M) and standard deviations (SD) of initial investment, mean investment and 1H-MRS metabolite levels per group: Cr, creatine; Glx, glutamate plus glutamine; NAA, *N*-acetylaspartate.

	Controls (n = 18)		Responsive (n = 21)		Resistant (n = 21)	
	*M*	*SD*	*M*	*SD*	*M*	*SD*
Initial investment	6.55	2.9	6.61	1.93	5.91	2.67
Mean investment	7.41	1.71	6.48	1.49	6.72	1.62
Glutamate/Cr	1.29	0.13	1.33	0.17	1.32	0.14
Glx/Cr	1.72	0.27	1.71	0.31	1.76	0.32
NAA/Cr	1.25	0.07	1.25	0.11	1.12	0.13
Choline/Cr	0.25	0.04	0.26	0.04	0.26	0.02
Myo-inositol/Cr	40.6	9.4	41.3	10.4	41.5	10.6

### Group effects of BOLD × glutamate interaction: treatment-resistant vs treatment-responsive

**Figure 2:**
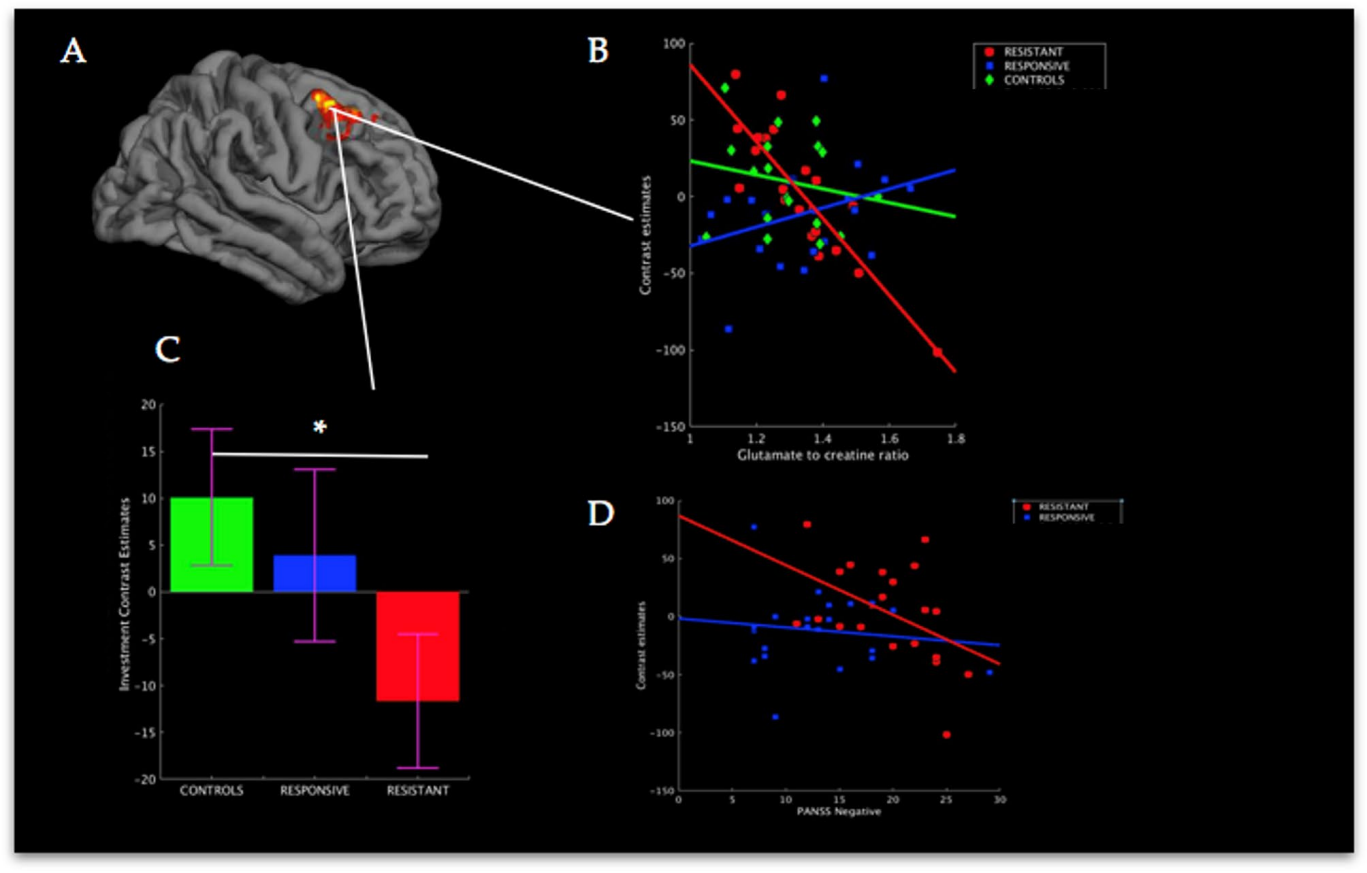
Glutamate interactions with BOLD signal from the resistant vs responsive contrast. (**A**) Right DLPFC cluster where the interaction between BOLD signal and glutamate during the investment phase was different between treatment-resistant and treatment-responsive patients, P < 0.001, whole brain-brain corrected (n = 702 voxels). (**B**) Association between BOLD signal extracted from cluster shown in panel A and glutamate to creatine ratio per group during investment. (**C**) Mean BOLD signal for per group from rDLPFC cluster, error bars represent standard error of mean. (**D**) Association between PANSS Negative and BOLD signal from rDLFPC. *Two-sample t-test, two-tailed, P < 0.05.

During the investment phase ACC glutamate levels in treatment-resistant patients significantly modulated activation in a right dorsolateral prefrontal cortex (DLPFC) cluster, corresponding to Brodmann Area 8 (BA8), compared to treatment responsive patients [MNI: x = 44, y = 14, z = 36, *Z*-score = 5.4, *P* < 0.001, whole-brain correction; Fig. 2A]; higher glutamate values were associated with signal decreases in this cluster [*R* = − 0.87, *P* < 0.001; Fig. 2B]. Mean signal from this cluster was significantly reduced for treatment-resistant patients compared to the control group [*T*(37) = 2.12, *P* < 0.05, two-tailed], reflecting a large effect size of *d* = 0.7 (Fig. 2C). The difference between treatment-resistant and treatment-responsive patients was not statistically significant, [*T*(40) = 1.33, *P* = 0.18], however, had a medium effect size of *d* = 0.42. No significant differences were found when comparing treatment-responsive patients and control participants [*T*(37) = 0.5, *P* = 0.6, effect size = 0.16). In terms of association with symptoms, signal reduction from this region correlated significantly with the severity of negative symptoms for treatment-resistant patients [*R* = − 0.47, *P* < 0.05], while no association was found for treatment-responsive patients [*R* = − 0.13, P > 0.2; Fig. 2D].

### Group effects of BOLD × glutamate interaction: treatment-resistant vs controls

**Figure 3:**
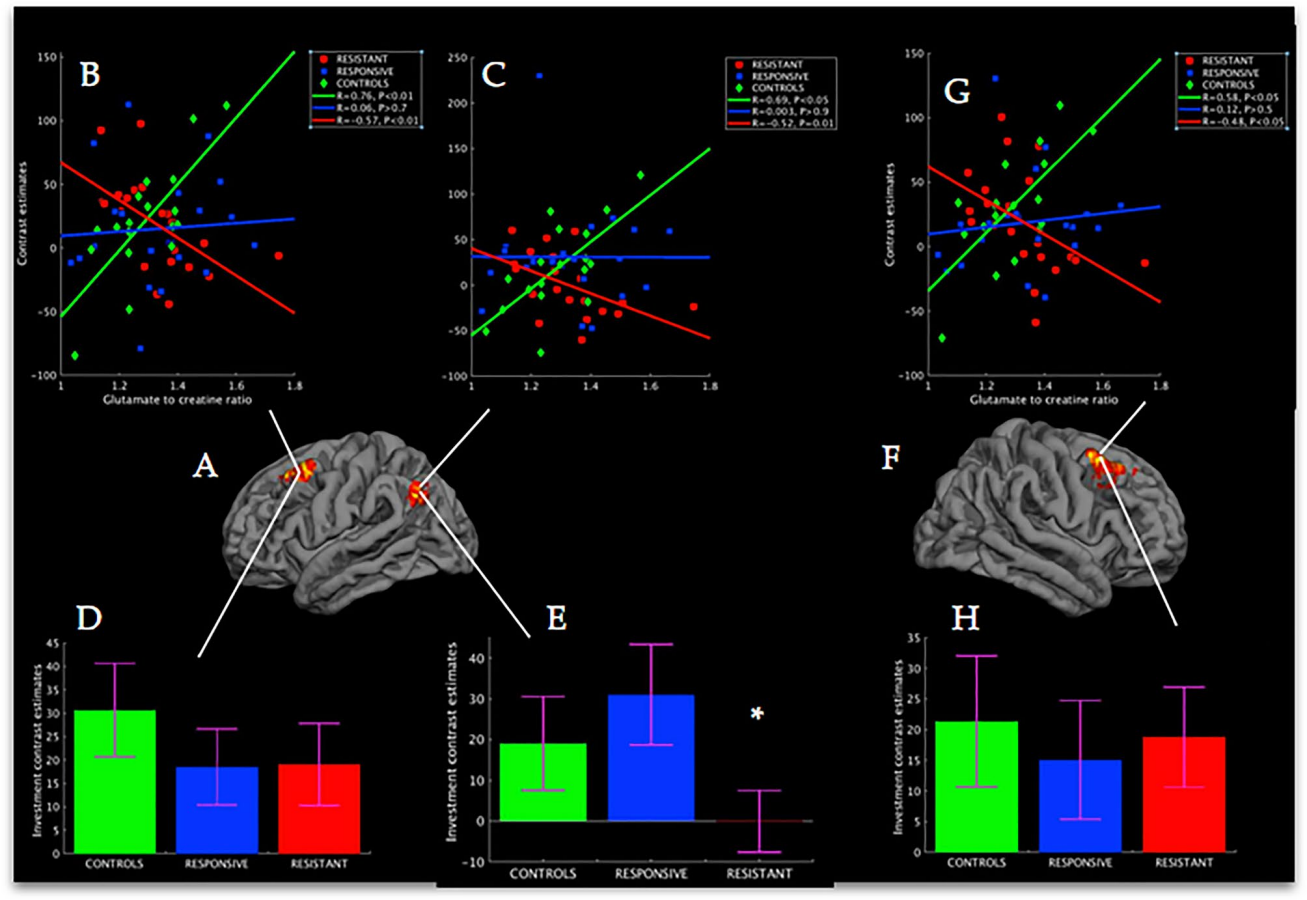
Glutamate interactions with BOLD signal from the resistant vs controls contrast. (**A**) Whole-brain corrected cluster for left DLPFC (P < 0.005, n = 1050 voxels) and PAC (P < 0.05, n = 726 voxels) where the interaction between BOLD signal and glutamate during the investment phase (n = 20 trials) was different between treatment-resistant and controls. (**B**) Association between BOLD signal extracted from lDLFPC cluster and glutamate per group during investment trials. (**C**) Association between BOLD signal extracted from PAC cluster and glutamate to creatine ration per group during investment trials. (**D**) Mean BOLD signal per group from lDLPFC cluster, error bars represent standard error of mean. (**E**) as in (**D**) for the left PAC cluster. (**F**) As in (**A**) for the right DLFPC cluster (P < 0.05, n = 801 voxels). (**G**, **H**) As in (**B**) and (**D**) for the rDLPFC cluster mean BOLD signal per group from rDLPFC cluster.

When treatment-resistant patients were directly compared to control participants, differential glutamatergic modulation was found in left DLPFC, corresponding to left Brodmann Area 8 [MNI: x = − 28, y = 30, z = 52, *Z*-score = 6.44, P < 0.001; Fig. 3A], right DLPFC, corresponding to right BA8 [MNI: x = 40, y = 12, z = 56, Z-score = 6.05, P < 0.05; Fig. 3F], and left lateral parietal association cortex (PAC) corresponding to BA39-BA40 [MNI: x = − 52, y = − 62, z = 32, Z-score = 5.81, P < 0.05; Fig. 3A). Increases in glutamate were associated with decreased activation across all regions in treatment-resistant patients; with increased activation in controls; and no change in treatment-responsive patients (Fig. 3B,C,G). When testing for group differences in mean activation within these clusters, treatment-resistant patients showed reduced signal compared to controls in the left PAC (Fig. 3E), while no differences were found in the DLPFC regions, Ps > 0.3 (Fig. 3D,H).

### Group effects of BOLD × glutamate interaction: treatment-responsive versus controls

No differences were found when directly comparing treatment-responsive patients with controls.

### Striatal region of interest analysis

**Figure 4:**
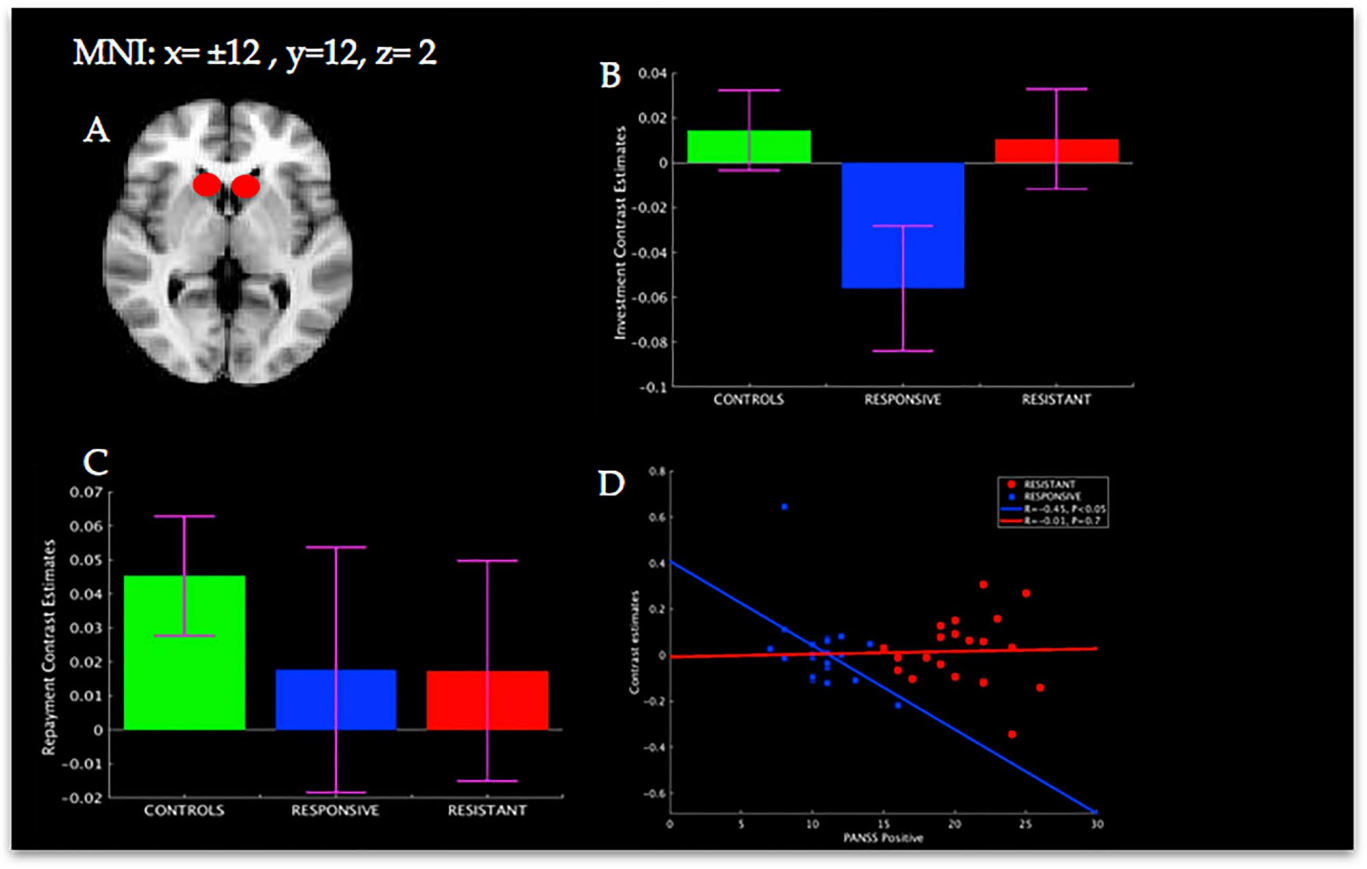
Anterior caudate mean signal per group and association with symptoms. (**A**) Red circles represent bilateral anterior caudate region of interest. (**B**) Mean BOLD signal per group from anterior caudate region of interest for the investment phase. (**C**) Mean BOLD signal per group from anterior caudate region of interest for the repayment phase. (**D**) Association between PANSS Positive and BOLD signal from anterior caudate region of interest. *Two-sample t-test, two-tailed, P < 0.05.

For the investment phase, treatment-responsive patients showed reduced caudate activations compared to control participants [*T*(37) = 2.05, P < 0.05, *d* = 0.67], and borderline significant reductions compared to treatment-resistant patients [*T*(40) = 1.9, *P* = 0.06, *d* = 0.6] (Fig. 4B). There was no difference between treatment-resistant patients and control participants [T(37) = 0.13, *P* > 0.5, d = 0.04]. No statistically significant differences were found for the repayment phase (Fig. 4C). For treatment-responsive patients, there was a significant correlation between striatal signal reductions and severity of positive symptoms during the repayment phase [*R* = − 0.45, *P* < 0.05], while no association was found for treatment-resistant patients [*R* = − 0.01, *P* = 0.7; Fig. 4D].

### Whole-brain fMRI effects

There were no significant main effects of group for either investment or repayment trials. The main effect of task in investment trials showed large bilateral prefrontal and premotor cortex clusters within Brodmann Areas 8 and 9 [k = 1328, *Z*-score = 9.70, MNI: x = − 54, y = 4, z = 36; k = 1328, *Z*-score = 5.57, MNI: x = − 48, y = 20 z = 26] as well as left occipital cluster within Brodmann Area 18 [k = 1099, *Z*-score = 5.83, MNI: x = − 10, y = − 94, z = − 10]. For the main effect of repayment trials there was a significant cluster extending from prefrontal and inferior frontal cortex within Brodmann Areas 8 and 44 [k = 444, *Z*-score = 4.90, MNI: x = − 48, y = 10, z = 32].

### Receiver operating characteristic curves analysis

As an exploratory analysis, we investigated the discriminative ability of the differential effects we found between treatment-resistant and treatment-responsive patients. We used as predictors ACC glutamate values, rDLPFC contrast estimates from the BOLD × glutamate patient group contrast and the caudate contrast estimates (shown Fig. 3A). Then we entered them in a logistic regression model to estimate binary probabilities of corresponding to treatment-resistant and treatment-responsive patients. Finally, the probability estimates from the logistic regression were used to compute the ROC curve. We found that these variables were associated with an area under the curve of 0.72, with an optimal operating point of 72% sensitivity and 77% specificity in differentiating treatment-resistant and treatment-responsive patients.

## Discussion

Our results provide insights for a plausible mechanism of why symptoms might be persistent in refractory patients despite apparently adequate antipsychotic medication treatment. In such individuals, glutamate specifically modulates processing in cortical substrates of the reward network, such as the DLFPC and PAC. These two regions play a key role in directing attention towards potentially rewarding events, monitoring reward expectations and updating the history of reward outcome, primarily through top-down NMDA signaling^23,24^. As glutamatergic indices measured by 1H-MRS are associated with NMDAR function^25^, this suggests a predominating NMDAR dysfunction in the DLPFC and PAC for those not responding to treatment. Notably, these differences appear to be quite robust, as they are revealed without an a priori regions of interest definition. We also found that in treatment-resistant individuals, glutamatergically modulated DLPFC activation was associated with greater rates of negative symptoms. This is consistent with evidence of poor antipsychotic response being associated with increased prodromal negative symptomatology^26^ and severe premorbid impairments in cognition and function^26,27^.

We found that treatment-responsive individuals show a reward processing dysfunction in the caudate, which was also associated with the severity of their positive symptoms. These results are consistent with a predominantly striatal dopaminergic dysfunction in patients who are responsive to antipsychotic medication. Caudate activation has been found to increase with more generous repayments in healthy participants^18^, while people with schizophrenia typically show significant signal reductions during repayments compared to healthy controls^19,28^—presumably modulated by dopaminergic dysfunction. Furthermore, we show that chronic treatment-resistant participants appear to have normal striatal reward signaling, consistent with previous evidence for normal dopaminergic function in treatment-resistant individuals (e.g.^7^). Notably, the activation pattern we found for the three groups mirrors that of presynaptic dopamine synthesis capacity reported in Demjaha et al.^7^ for whole striatum and its subdivisions. Putatively, the signal decreases we observe for responsive patients in the task we used could be probing reward processing modulated by dopaminergic function. If so, it also corroborates the notion of non-dopaminergic deficits in treatment-resistant patients.

Notably, there were no significant differences between groups in ACC glutamate, despite previous evidence for elevated glutamate in chronic treatment-resistant patients^9^. In addition, no whole-brain BOLD signal group differences were found for either investment or repayment trials. However, our results show strong interactions between Glu/Cr and BOLD signal that highlight the strength of multimodal imaging in investigating antipsychotic treatment response. The integration of neurochemical and functional imaging modalities revealed highly significant associations between glutamate and reward processing differentially in treatment resistant and treatment-responsive patients, even in the absence of group differences in each modality separately.

Taken together, our data reveal a plausible neurobiological mechanism underpinning a non-dopaminergic, but rather primarily glutamatergic dysfunction, which modulates aberrant reward processing in treatment-resistant patients. Optimal reward processing and learning is predicated by the intact function of both subcortical substrates, such as the striatum and the amygdala, but also cortical substrates, such as prefrontal and parietal cortex^11,29–32^. Our results suggest that treatment-responsive individuals have a primarily dopaminergic dysfunction that results in aberrant reward processing, and it is attenuated by dopamine antagonists. Those who are treatment-resistant appear to have a primarily glutamatergic dysfunction that modulates key cortical nodes of the reward system. Dopamine antagonist antipsychotic drugs do not directly normalize aberrant reward processing in these regions or can have a small bottom-up effect given the direct connectivity the striatum with the prefrontal and parietal cortex. This may explain why some treatment-resistant patients can report a transient attenuation of their symptoms with the initial instigation of medication. However, as aberrant reward processing in treatment-resistant patients is not primarily caused by dopaminergic dysfunction, in the longer-term, dopaminergic antipsychotic medication fails to normalize glutamatergic dysfunction and correct reward processing deficits.

The mechanism we describe provides a possible explanation for the increased efficacy of clozapine, including in those previously determined to be refractory to treatment. Clozapine shows only moderate affinity to striatal D2 receptors^33^, but increased binding in a number of non-dopaminergic receptor sites and prominently binds to NMDAR^34^. It also shows specificity over haloperidol in modulating partial NMDA agonists glycine and serine^35^ which are perturbed in treatment-resistant patients^36,37^. Thus, a potentially mechanism of action is that it normalizes glutamatergic/NMDAR function and corrects aberrant reward signaling. Indeed, recent evidence suggests that clozapine attenuates glutamate in treatment-resistant patients (McQueen et al., 2021). This study, however, did not measure BOLD responses in reward processing in association with glutamatergic changes induced by clozapine, which needs to be addressed by future research.

The findings of the present study provide evidence for the relevance of NMDA/glutamate models of schizophrenia specifically to antipsychotic treatment-resistance. Current models postulate complex dopamine-glutamate interactions to explain excessive striatal dopamine levels, with no apparent consensus in the primacy of one system over the others^38,39^. However, these cannot account for the development and persistence of psychotic symptoms in treatment-resistant individuals assuming normal dopamine levels. Our results show that reward processing can be disrupted by a glutamatergic modulation of key cortical substrates of the reward network (primarily through the DLPFC and PAC), without any apparent aberrant striatal signaling. This raises the need for updated pharmacological models of schizophrenia where psychosis can arise from causes not directly linked to abnormal dopaminergic function.

In terms of limitations, the cross-sectional design could not determine causal relationships and timing in the differentiation of cortical and sub-cortical reward substrates in treatment-resistant and treatment-responsive individuals. This question could be potentially resolved with a prospective design, measuring these effects in medication-naïve clinical populations, and repeating such measurements if and when treatment resistance has been determined. Our results show that glutamatergic modulation of aberrant reward processing appears to be a stable neurobiological trait for chronically-unwell treatment-resistant individuals. 1H-MRS glutamate measures are not a direct index of NMDAR function. Direct imaging of NMDAR (but also other glutamate receptors that can be implicated^39^) is challenging and appropriate methods are still largely under development. A recent study provided evidence for NMDAR hypofunction in the hippocampus for first-episode schizophrenia patients^40^. Once NMDAR imaging is further established can provide more accurate indexes of glutamatergic neurotransmission that will further elucidate its role in psychosis and treatment-response. One potential confound is a selection bias given the compensation for participation in the study, given the important role of both impaired reward processing and poorer socio-economic circumstances experienced by patients. However, within the study, the key comparisons between patients should not be influenced by this and indeed the healthy volunteer group are from the same community as the patients, matching broader macro socio-economic factors. Finally, the discriminant analysis we conducted had a relatively small sample size to allow for further analysis on the predictive value of the variables we used. Nevertheless, it demonstrated that aberrant reward processing in dissociable neural substrates can have potential diagnostic use. However, it requires future validation with an independent sample, as well as further testing of the classifier in first-episode patients to predict their response.

In conclusion, our data show that individuals with schizophrenia show variability in the neurochemical perturbations that contribute to the reward processing deficits, and that these can differentiate treatment-resistant from non-treatment-resistant individuals. What causally determines this is yet to be established, and an interplaying multitude of genetic, neurodevelopmental, behavioral and environmental factors can be put forward^41,42^.

## Data Availability

The primary data are available for further scrutiny and analysis as required.
