# Author Correction: Neuroimaging glutamatergic mechanisms differentiating antipsychotic treatment-response

**DOI:** 10.1038/s41598-023-38413-1

**Published:** 2023-07-14

**Authors:** Elias D. Mouchlianitis, Lucy D. Vanes, Derek K. Tracy, Anne-Kathrin Fett, Daniel Joyce, Sukhi S. Shergill

**Affiliations:** 1 Institute of Psychiatry, Psychology and Neuroscience, De Crespigny Park, London, SE5 8AF UK; 2 School of Psychology, University of East London, Water Lane, Stratford, London, E15 4LZ UK; 3 West London NHS Trust, London, UB2 4SD UK; 4 Department of Psychiatry, University College London, London, W1T 7BN UK; 5 Department of Psychology, City, University of London, London, EC1V 0HB UK; 6 Oxford Health NHS Foundation Trust, Oxford, OX4 4XN UK; 7 Kent and Medway Medical School, Kent, CT2 7FS UK

Correction to: *Scientific Reports* 10.1038/s41598-022-26702-0, published online 02 June 2023

The original version of this Article contained errors. The spelling of the given name and the affiliation of the author Anne-Kathrin Fett were incorrectly given as ‘Anne-Katherin Fett’ and ‘Department of Educational and Family Studies and LEARN! Research Institute, Vrije Universiteit Amsterdam, Amsterdam, The Netherlands’, respectively. The correct affiliations are listed below.

Institute of Psychiatry, Psychology and Neuroscience, De Crespigny Park, London, SE5 8AF, UK

Department of Psychology, City, University of London, London, EC1V 0HB, UK

The original Article has been corrected.

